# *Chlorella vulgaris* as a food substitute: Applications and benefits in the food industry

**DOI:** 10.1111/1750-3841.17529

**Published:** 2024-11-18

**Authors:** Chiao‐An Wang, Helen Onyeaka, Taghi Miri, Fakhteh Soltani

**Affiliations:** ^1^ School of Chemical Engineering University of Birmingham Birmingham UK

**Keywords:** *Chlorella vulgaris*, dietary, food substitute, health benefits, nutritional value

## Abstract

*Chlorella vulgaris*, a freshwater microalga, is gaining attention for its potential as a nutritious food source and dietary supplement. This review aims to provide a comprehensive discussion on *C. vulgaris*, evaluating its viability as a food substitute in the industry by exploring the nutritional value and application of *C. vulgaris* in the food industry. Rich in protein, lipids, carbohydrates, vitamins, and minerals, *Chlorella* offers substantial nutritional benefits, positioning it as a valuable food substitute. Its applications in the food industry include incorporation into smoothies, snacks, and supplements, enhancing the nutritional profile of various food products. The health benefits of *Chlorella* encompass antioxidant activity, immune system support, and detoxification, contributing to overall well‐being. Despite these advantages, the commercialization of *Chlorella* faces significant challenges. These include variability in antibacterial activity due to strain and growth conditions, high production costs, contamination risks, and sensory issues such as unpleasant taste and smell. Additionally, *Chlorella* can accumulate heavy metals from its environment, necessitating stringent quality control measures. Future prospects involve improving Chlorella strains through genetic manipulation to enhance nutrient content, developing cost‐effective culture systems, and exploring advanced processing techniques like pulsed electric fields for better digestibility. Addressing sensory issues through flavor‐masking strategies and employing environmental management practices will further support *Chlorella's* integration into the food industry. Although *C. vulgaris* shows great potential as a nutritious food ingredient, overcoming existing challenges and optimizing production methods would be crucial for its successful adoption and widespread use.

## 1 INTRODUCTION

*Chlorella vulgaris* is a green unicellular microalgae of the order *Chlorococcales*, family Oocytaceae, and genus *Chlorella*. It has a green hue because of the presence of chloroplast within it (Coronado‐Reyes et al., 2020). *Chlorella vulgaris* ranges in size from 2 to 10 µm in diameter and is spherical, subspherical, or ellipsoid and lacks flagella (Champenois et al., 2015). They exist as single cells or can form clones up to a size of 64 cells. *Chlorella vulgaris* has only one cup‐shaped chloroplast with or without pyrenoids, which contain the starch grains. Since *C. vulgaris* is a nonmotile microalgae, it reproduces through asexual autospores (Safi et al., 2014), which is through the division of the mother cell into 2–32 autospores or daughter cells. The mother cell wall breaks after the formation of autospores and the mother cell's remains are consumed by the daughter cells through autosporulation (Ru et al., 2020; Yamamoto et al., 2014). They are frequently found in freshwater, marine, and terrestrial habitats and have higher photosynthetic efficiency and the competency of faster growth in both autotrophic, mixotrophic, and heterotrophic manners (Panahi et al., 2016; Tomaselli, 2004). These microalgae contain, besides chlorophyll, intracellular proteins, carbohydrates, lipids, vitamin C, β‐carotenes, and B vitamins (B1, B2, B6, and B12). And for this reason, it is used for the preparation of food supplements, cosmetics, clinical treatments, and the neutralization of heavy metals in industrial wastewater (Coronado‐Reyes et al., 2020).

There has been a growing tendency to seek innovations in regard to the availability of food especially in the recent past as a result of environmental issues, population increase, and the need to improve food security. Traditional methods of farming are devastating to the environment due to the high water usage, greenhouse gas emissions, and land degradation. In turn, there are rising requirements for stable and adequate food sources in addition to sustainable food sources (Wani et al., 2024). *Chlorella vulgaris* has emerged as the most effective for resource usage. Compared to other crops, *C. vulgaris* can be produced in a controlled environment and does not need vast amounts of land and water (Al‐Hammadi & Güngörmüşler, 2024). For this reason, it has a fast‐growing cycle and high biomass output and can be used in commercial production. *Chlorella vulgaris* also contributes to environmental sustainability in carbon dioxide sequestration and wastewater bioremediation (Rosales‐Aguado et al., 2024). Consumers are increasingly becoming conscious to their health, which is why nutrients with clear functional health benefits are popular among consumers (Rani et al., 2018). According to Rzymski et al. (2019), *Chlorella* taken daily with an intake dose of 3–10 g/day has health benefits to the human body. Over the years, *C. vulgaris* has been incorporated into many foods and food products due to its rich nutritional value, and its putative health benefits include its antioxidant activity, ability to boost the immune system, and detoxification properties (Rani et al., 2018). *Chlorella vulgaris* has interested multiple researchers for its utilization in the development of dietary supplements, functional foods, and nutraceuticals; hence, strengthening its position and probable influence on the future trends of the food industry (Surasani et al., 2024).

This review aims to provide a comprehensive discussion on *C. vulgaris* and its ability as a food substitute in the food industry. It will discuss the nutritional value of *C. vulgaris* and how it is better than conventional foods. This review shall also examine the uses of *C. vulgaris* in food products, supplements, functional foods, and nutraceuticals. It would assess the benefits of *C. vulgaris* cultivation on the environment such as its contribution in carbon capture and wastewater treatment. This review aims to investigate the health benefits of *C. vulgaris* for consumers to enhance their health standards. It would cover the issues concerning the mass production and industrialization of *C. vulgaris* and the future possibilities of its application in human diets.

## 2 NUTRITIONAL CONTENT OF *Chlorella vulgaris*

*Chlorella vulgaris* has been used as a food item and is well known for its functional macronutrient and micronutrient components (Table 1), which include protein, omega‐3 polyunsaturated fatty acids, polysaccharide vitamins, and minerals (Panahi et al., 2016).

**TABLE 1:** Nutritional content of *Chlorella vulgaris*.

Nutrient category	Nutrient	Content‐range (% of dry weight)	Function	Source
**Proteins**	Total protein	43%–58%	Growth, repair, and maintenance of cells	Safi et al. (2014)
**Lipids**	Total lipids	5%–58%	Energy and carbon reservoir	Safi et al. (2014)
	Neutral lipids (mono‐, di‐, and triacylglycerol)	Varies	Stored in cell organelles	Garcia (2012)
	Polar lipids (phospholipids and glycolipids)	Varies	Predominant in cell wall and organellar membranes	Garcia (2012)
**Carbohydrates**	Total carbohydrates	12%–55%	Energy and carbon reserves	Choix et al. (2012)
	Starch	Major component	Energy reserve (amylose and amylopectin)	Choix et al. (2012)
	Cellulose	Present	Structural component in cell wall	Choix et al. (2012)
**Vitamins**	Vitamin A	Present	Vision, immune function	Maruyama et al. (1997), Safi et al. (2014), Yeh and Chang (2012)
	Vitamin B	Present	Energy metabolism	Maruyama et al. (1997), Safi et al. (2014), Yeh and Chang (2012)
	Vitamin C	Present	Antioxidant, immune support	Maruyama et al. (1997), Safi et al. (2014), Yeh and Chang (2012)
	Vitamin E	Present	Antioxidant, skin health	Maruyama et al. (1997), Safi et al. (2014), Yeh and Chang (2012)
**Minerals**	Calcium	Present	Bone health, muscle function	Yusof et al. (2011)
	Potassium	Present	Electrolyte balance, nerve function	Yusof et al. (2011)
	Magnesium	Present	Muscle function, nerve function	Yusof et al. (2011)
	Zinc	Present	Immune function, wound healing	Yusof et al. (2011)

### 2.1 Proteins

Proteins are present in large amounts in microalgae and range from 50%–70% of their structure depending on the specific type of species. They help in the growth of cells, repair, and maintenance. *Chlorella vulgaris* contains 43%–58% of the total protein content, of which its dry weight depends on the growth condition present (Safi et al., 2014). For microalgae, about 20% of these are fixed to the cell wall, 50% evenly distributed within the cell, while the remaining 30% of the proteins are mobile, whereby they move in and out of the cell wall. The total number of proteins varies between 12 and 120 kDa (kilodaltons), of which the majority of them fall within the range of 39–75 kDa. *Chlorella vulgaris* is important at the nutritional level because in autotrophic or heterotrophic media, they have a possibility to synthesize essential and nonessential amino acids required by human beings. Each of these components of the growth media, proteins and amino acids, can be separated by means of using alkaline solutions, ultrafiltration, or precipitating the proteins with trichloroacetic acid or 0.1 N hydrochloric acid (Safi et al., 2014). Among the amino acids that have been characterized in the growth kinetics of *C. vulgaris* are aspartic, glutamic acid, threonine, serine, glutamic acid, glycine, alanine, cysteine, valine, methionine, isoleucine, leucine, tyrosine, phenylalanine, histidine, lysine, arginine, tryptophan, ornithine, and proline, where glutamic acid is synthesized in large quantities, followed by aspartic acid with a concentration of 13.7 g/100 g of protein and 10.94 g/100 g of protein, respectively (Ursu et al., 2014). Within the composition of the amino acids identified in the biomass of this microalgae are some essentials such as histidine, isoleucine, leucine, lysine, methionine, phenylalanine, threonine, and valine but not tryptophan (Mohamed et al., 2013).

### 2.2 Lipids

Lipids are crucial structures in the metabolic and growth processes of microalgae because they act as energy and carbon storage (Kottuparambil et al., 2019). The lipids that are present in microalgae can be divided into neutral and polar lipids. Polar lipids are phospholipids and glycolipids produced by chloroplasts and found in abundance in cell wall and organellar membranes, while neutral lipids such as mono‐, di‐, and triacylglycerol are kept in the cell organelles (Garcia, 2012). The lipid content of microalgae has been identified to be between 2% and 77% based on their species and the growing conditions. *Chlorella vulgaris* has been noted to be between 5% and 58% of dry weight (Safi et al., 2014). *Chlorella vulgaris* obtain more lipid content (60%–68%) when it is grown under mixotrophic conditions (Yeh & Chang, 2012).

### 2.3 Carbohydrates

Carbohydrate sources like starch, glucose, sugar, and polysaccharides are generally employed for energy and carbon storage in microalgae (Choix et al., 2012). Starch, which is the most abundant form of polysaccharides, found the chloroplast level in *C*. *vulgaris* is made of amylose and amylopectin. Polysaccharide cellulose is present in the cell wall of *C. vulgaris* with glycosidic bond of β1‐3. Cellulose in *C. vulgaris* can be used as an input for the preparation of functional foods to enhance human health (Coronado‐Reyes et al., 2020). The total carbohydrate value in *C. vulgaris* can be between 12% and 55% dry weight provided the culture is under unfavorable conditions, particularly in a situation of limited nitrogen (Choix et al., 2012). The cell walls of the microalgae consist of rhamnose, galactose, glucose, xylose, arabinose, and mannose, of which rhamnose dominates the entire arrangement (Ho et al., 2011).

### 2.4 Vitamins and minerals

Vitamins are organic compounds that are necessary for organisms in very small amounts for the maintenance of proper growth and nourishment. Vitamins can be divided into vitamins B and C known as the water‐soluble vitamins and A, D, E, and K vitamins known as the fat‐soluble vitamins. Vitamins have to be consumed through diet since the human body is incapable of producing all these vitamins on its own. *Chlorella vulgaris* has vitamins A, B, C, and E (Maruyama et al., 1997; Safi et al., 2014; Yeh & Chang, 2012). Minerals are inorganic substances that are necessary in the body for proper functioning and overall health. *Chlorella vulgaris* is a good source of calcium, potassium, magnesium, and zinc (Yusof et al., 2011). Due to the presence of all these vitamins and minerals, *C. vulgaris* has been widely used to provide micronutrients to the human body.

## 3 PRODUCTION AND CULTIVATION OF *Chlorella vulgaris*

Different cultivation methods have been developed to give rise to different strains of *C. vulgaris*. The most typical way of increasing growth conditions is the culture of autotrophic growth (Elalami et al., 2021), which produces organic *C. vulgaris*, referred to as C‐Auto. Another economically feasible technique for the cultivation of *C. vulgaris* is heterotrophic growth, thus leading to heterotrophic *C. vulgaris* (C‐Hetero) (Barros et al., 2019). But the organoleptic characteristics of C‐Auto and C‐Hetero, which include strong color, taste, and smell, may reduce its use in numerous products (Lafarga, 2019).

*Chlorella vulgaris* can also be cultivated using open and closed cultivation methods. Open systems such as raceways and ponds are the simplest and most popular type, as the construction and functioning of such structures are inexpensive and fast but long‐lasting. Essentially, they are large vessels in which the microalgae are contained. It is permitted to grow under environmental conditions. They are simply maintained under conditions such that they flow or move in ways that are in contact with nutrients within the media and do not settle at the bottom of the tanks. Among the limitations that these systems present, the cells are not constantly in contact with light, which limits the efficiency of photosynthesis; the culture medium evaporates; a large size is necessary for the construction of the systems; and there is a risk of being infected by other organisms. Due to these limitations, they are only suitable for resistant species (de Godos et al., 2009).

Closed systems, such as photobioreactors and fermenters, on the other hand, provide more control over the culturing conditions than the open systems. In addition to that, they have higher biomass yield when polluting factors are avoided and the light source is artificial, solar, or both at the same time. Culture systems of closed systems can be bubble columns, airlift columns, agitated, conical, and tubular helical tanks. The main advantage of this system is that there is rigorous control of the experimental conditions, ease in sterilization of the cultures, and simple acclimated and minimized photo‐oxidation compared to the open systems, but these photobioreactors are relatively expensive than the open systems (Kunjapur & Eldridge, 2010).

Estevam et al. (2022) cultivated *Chlorella* sp. in open and closed lab‐scale systems. Their study found out the density of the open pond culture achieved the highest specific growth rate (0.52 L/day) of 5.06 × 10^7^ cells/mL overall cell density and biomass concentration of 1.30 mg/mL. In this condition, it was observed that the level of pigment content was comparatively higher where the chlorophyll‐a was 13 µg/mg, chlorophyll‐b 15 µg/mg, and carotenoids 2 µg/mg. Also, the percentage of the lipid content has been shown to have a much higher growth as compared to the initial level of 17% and reaching 35% under limited nitrogen conditions with high phosphorus content. On the other hand, the protein percentage improved as the levels of both nutrients increased, reaching the highest level of 60%. However, the percentage of carbohydrates reduced from 32% to 13% while phosphorus levels reduced (Estevam et al., 2022).

## 4 *Chlorella vulgaris* AS A FOOD SUBSTITUTE

**FIGURE 1:**
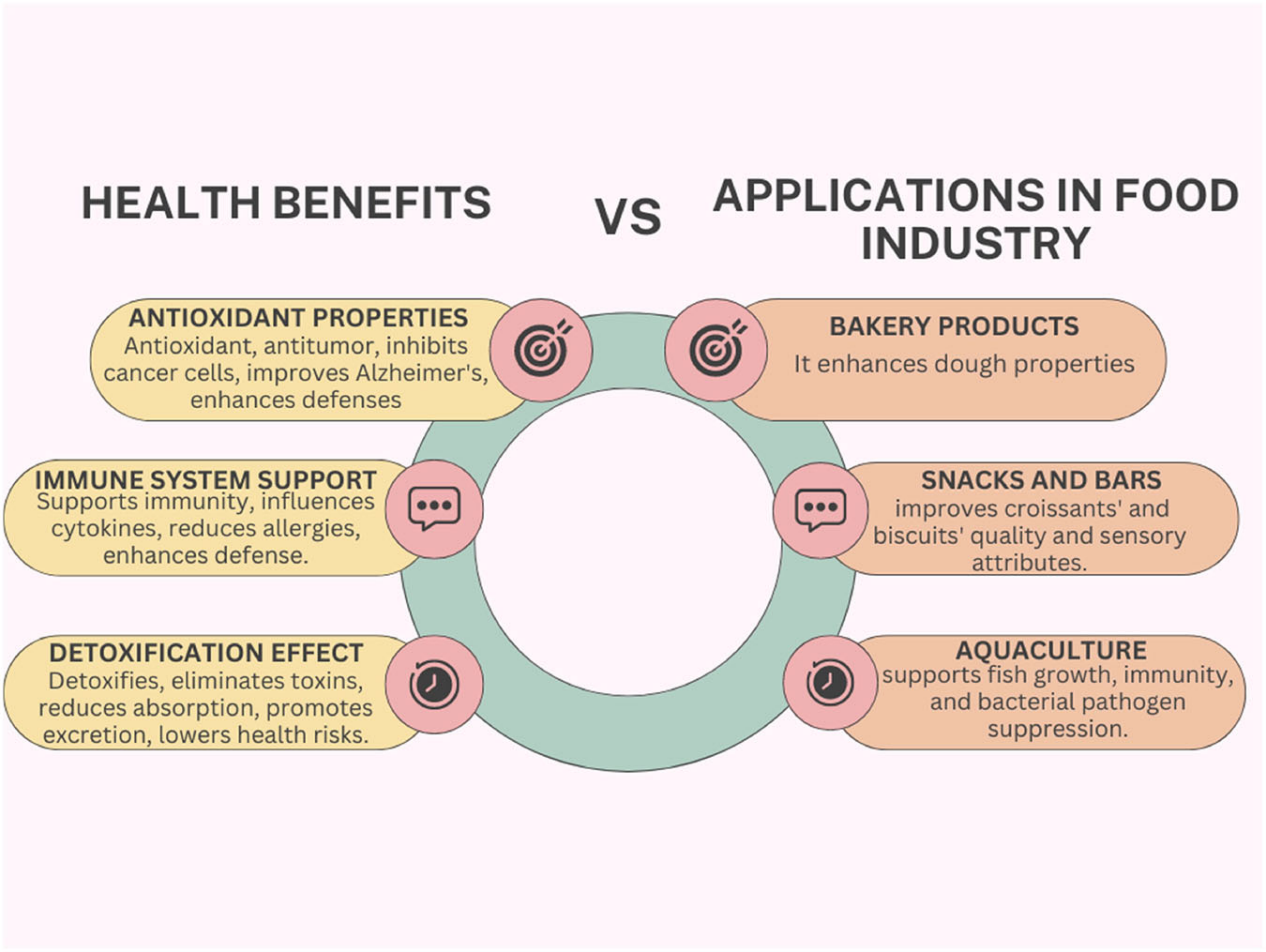
Health benefits and applications of *Chlorella vulgaris* in the food industry.

*Chlorella* has been used as a food supplement for many years. *Chlorella*, being a popular supplement, is being consumed in its powder and tablet forms, as it has the natural capacity to bind toxic metals including mercury and expel them from the body. It has many health benefits and applications in the food industry, as seen in Figure 1. Considering this, some research has been performed about the prospects of developing food products from *Chlorella* biomass. Cereals and sugary products such as croissants, pasta, yoghurt, and cookies (Beheshtipour et al., 2013; Fradique et al., 2010; Shalaby & Yasin, 2013) have also been fortified with *Chlorella*. The incorporation of *Chlorella* biomass into those food products has generated aesthetically pleasing and market‐innovative products with progressively higher functional ingredients especially essential amino acids, antioxidants, polyunsaturated fatty acids, and vitamins. Furthermore, *Chlorella* addition may also enhance the survival rates of probiotics in fermented dairy products such as yogurt (Widyaningrum & Prianto, 2021). *Chlorella* can enhance the nutrition and organoleptic characteristics of food products. There is a difference in the physical properties of croissants with the incorporation of *C. vulgaris*, as it improved the looks, taste, and texture (Shalaby & Yasin, 2013). These results correlate well with those obtained in a study conducted by Fradique et al. (2010), where the panelists favored pasta prepared from *C. vulgaris*, particularly those at concentrations of 2%, because of the novelty and appeal to the appearances. It is also evident from the results that cookies with the addition of *C. vulgaris* have higher textural characteristics. This is explained by the biochemical characteristic of *Chlorella*’s high protein content, which directly influences the outcome. The protein molecules strengthened the dough system and, in this way, influenced the water absorption leading to an augmentation of cookies’ firmness (Gouveia et al., 2002).

### 4.1 Applications in the food industry

Due to high protein content and the presence of essential nutrients, scientists took interest in *C. vulgaris* for its low cost, high‐quality edible, and cheap food source during World War I (Belasco, 1997). At the end of the Industrial Revolution, *C. vulgaris* was taken as a food supplement, especially in Germany, China, Japan, the United States, and Europe, and still today it is taken in the same manner (Gouveia et al., 2002; Liu & Chen, 2014). As for other applications in the food industry, the high protein of *C. vulgaris* has facilitated its application as an emulsifier (Raymmundo et al., 2005). Some polysaccharides including agar and alginates from *C. vulgaris* are used for thickening and gelling in foods (Brennan & Owende, 2010), while natural colorants from *C. vulgaris* enhance the stability of color parameters in food products and good texture (Gouveia et al., 2002).

#### 4.1.1 Bakery products

The effect of incorporating *C. vulgaris* into wheat flour dough was investigated in a study by Graça et al. (2018) based on dough rheology and bread quality characteristics. Microalgae contents in the dough formulation included 1.0, 2.5, and 5.0 g/100 g of wheat flour. The study established that up to 3.0 g of microalgae biomass improved the rheological properties of the dough and viscoelastic gluten networks. However, when the microalgae amount increased, it caused a detrimental effect on the dough rheology, textural and sensorial characteristics of bread, and enhanced rate of staling. Graça et al. (2018) also noted that the biomass addition did not affect the kinetics of yeast fermentation or the time for the commencement of the fermentation process (Graça et al., 2018). Marzec et al. (2023) established that the type of flour influences both the experimental raw dough rheology, the texture, and the microstructure of the muffins with the addition of microalgae (*C. vulgaris*). Although the characteristics of the dough remained acceptable, some differences were noticed. The addition of spelt flour resulted in a more viscous, less cohesive, and less springy dough as compared to wheat flour. The incorporation of 1.5% microalgae into a combination of spelt and wheat flour affected the rheological properties of a muffin dough positively by increasing the viscosity, and this created a fine and porous microstructure with a good crumbly texture. The use of wheat flour dough with the addition of 1.5% microalgae led to changes in the consistency and porosity of the finished product. The number of closed pores influenced the texture of the muffins. The microstructure of the samples was also a major concern or factor that affected the texture of the muffles in a major way. Consumers’ acceptability ratings of all the muffin types for appearance, taste, and overall acceptability were high (Marzec et al., 2023).

Maiti et al. (2024) incorporated *C. vulgaris* microalgae biomass at different concentrations (0.6%, 0.8%, and 1.0%) in 100 grams of different blends of foxtail millet and little millet flour to evaluate how these different flour blends and microalgae concentrations affected the physicochemical properties of millet pretzels such as protein content. A semi‐trained panel also assessed the sensory characteristics, which included color, appearance, taste, flavor, texture, and the overall quality. Using microalgae biomass at 1% concentration in pretzels was beneficial, enriching the product proteins (Maiti et al., 2024). However, pretzels with 0.8% microalgae showed better sensory value, signifying better taste and overall sensory acceptability. Dündar et al. (2023) found that cookies’ protein, ash, and fat levels increased significantly with *C. vulgaris* microalgae content, while carbohydrate content had no significant changes.

#### 4.1.2 Snacks and bars

The study by Shalaby and Yasin (2013) found that the addition of *C. vulgaris* biomass enhanced the quality indices of imitation cheese‐filled croissants as compared with the control sample. The chemical composition and staling rate of croissants were stable after the addition of cheese enriched with microalgae into the formulation. Shalaby and Yasin (2013) also identified that the addition of freshly prepared imitation cheese in croissants led to the improvement of sensory quality characteristics when croissants were filled with biomass of *C. vulgaris* in concentrations of 2% and 3%. More specifically, the respective impacts were identified in taste, texture, and eating quality, rendering the products more attractive to consumers. Gouveia et al. (2002) found that the attributes related to the biscuits’ texture were enhanced, and the color and texture remained constant for 3 *Chlorella*‐infused months in biscuits. When the biomass content was modified from 1.0% to 3.0%, the biscuits modified their brownish color to a green and duller color (Gouveia et al., 2002).

#### 4.1.3 Aquaculture

In the commercial aspect, *C. vulgaris* is one of the widely used microalgae found in aquaculture. Some of the literature validated its positive effects on feeding, protection of immunity, water treatment, reduction of stress, disease resistance of fish, and the ability to act against bacterial communication when properly applied (Ahmad et al., 2020). However, although *C. vulgaris* has been said to have positive impacts when it is added to diets, it was reported to have negative impacts on diets at higher inclusion rates. Its cell wall can become more rigid and might hinder the entry of digestive enzymes to intracellular constituents for proper digestion and absorption (Ahmad et al., 2020).

Microalgae species like *Chlorella* sp. are usually applied in finfish hatcheries primarily as feed for rotifers or *Artemia* sp. (Siddik et al., 2024). By using this method, nutrients such as fatty acids, amino acids, carotenoids, vitamins, and minerals are accumulated in the body of the zooplanktons from the microalgae, enhancing the nutritional value of the zooplanktons. Zooplankton that is enriched with microalgae is favorable in the provision of enrichment tools for fish and shellfish larvae (Siddik et al., 2024). For instance, the maximum growth and survival rates of *Betta splendens* were observed by a fish‐fed copepod enriched with *Chlorella* sp. *Chlorella* sp. can allow replacement of fish oil and fish meal in diets for better growth and meat quality, and for immune stimulations and pigmentation in the various species of fish (Alagawany et al., 2021). It supports growth in several species, such as carp, tilapia, Asian seabass, and Nile tilapia. Apart from growth enhancement, *Chlorella* microalgae have been subsequently manifested in the enhancement of the immune system in different types of fish. More precisely, *Chlorella* sp. can also promote phagocytosis activity, the number of white blood cells, and the relative expression of cytokine genes that involve a critical component of immune response (Alagawany et al., 2021). Also, in the context of aquaculture, there is conventional knowledge that the survival rate of the fish larvae provides a dramatic increase when fed with live feeds enriched with the carotenoid pigments, the rotifers, and *Artemia* sp. Some of these feed directly on feed ingredients and include poultry and swine, while others feed on fish meal and other feed supplements and include fish, crustaceans, butterflies/moths, and copepods (Alagawany et al., 2021).

In vitro and in vivo investigations showed the possibility of using *Chlorella* to eliminate bacterial fish pathogens in aquaculture. Thus, *Chlorella* has bioactive compounds such as polysaccharides, proteins, and lipids that have been reported to suppress bacterial growth and biofilm (Aly et al., 2024). The impact of *Chlorella* also depends on the growth conditions in treating bacterial fish pathogens. It was established that the content of specific nutrients present in the growth medium can affect the synthesis of the bioactive compounds and *Chlorella's* antibacterial effect. Martinez and Orus (1991) have revealed that *Chlorella* densities enriched with glucose contained higher amounts of bioactive substances and a higher antibacterial activity effect on *Vibrio parahaemolyticus* as compared to *Chlorella* densities cultivated with no glucose. It was also found that the growth temperature and pH of *Chlorella* can also influence its antibacterial activity. The administration route of *Chlorella*‐based treatments can also influence the effectiveness of the treatments for bacterial fish pathogens. *Chlorella* can be ingested via water in edible form or mixed into water to bathe the fish, and depending on the species of fish and the type of bacterial pathogen, the two methods of administration can be effective in fighting the bacterial infection. According to the study conducted by El‐Habashi et al. (2019), the study successfully showed that *Chlorella* feeding enhanced the survival rate of the juvenile tilapia infected with *Aeromonas hydrophila* and decreased the rate of bacterial loads. On the other hand, J. Li et al. (2023) established that immersion treatment with *Chlorella* extract was more effective compared to oral administration in eradicating bacterial loads and enhancing the immune response of the grass carp infected with *A. hydrophila*.

### 4.2 Health benefits and functional properties

Over the previous years, individuals have realized that foods have a direct impact on their health. As a result, there is a growing research concern about products with natural origin and with high biological value components. In this case, *Chlorella* qualifies to be used as functional foods, which means products that contain certain substances that have benefits to the body other than being sources of nutrients in the body (Wells et al., 2017). Thus, *Chlorella* intake can beneficially affect human health and increase life quality causing a decrease in the risk of diseases (Matos et al., 2017).

#### 4.2.1 Antioxidant properties

Hot‐water extract of *C. vulgaris* (Konishi et al., 1985) and acetone extract (Tanaka et al., 1990) are said to exhibit antitumor properties. An aqueous extract of *Chlorella is* rich in antioxidants and interferes also with cell proliferation in human hepatoma cells (L. C. Wu et al., 2005). Three carotenoids identified as antheraxanthin, zeaxanthin, and lutein isolated from *Chlorella* cells were seen to have a profound negative effect on the human colon cancer cells (Cha et al., 2008). These findings indicate that it is the additive effect of several bioactive compounds in *Chlorella* that contribute to its antitumor activity. Another study by Romos et al. (2010) expressed that *Chlorella* can affect immunomyelopoietic activity and negate the suppressive effect of the tumor on cytokines and other cell assignments in tumor‐bearing mice. It has been found that a 63 1‐kD antitumor glycoprotein was present in the culture supernatant of *C. vulgaris* strain CK22 (Noda et al., 1996), and its chemical nature and antitumor activity have already been investigated and may partly be due to the presence of the aforementioned glycoprotein (Hasegawa et al., 2002).

Alzheimer's disease is a progressive disorder that affects the neurological cells in the brain. The erythrocytes of Alzheimer's disease patients are also recognized to be in an over‐oxidized state (Selkoe 2001). α‐Tocopherol and carotenoids including lutein are another major lipophilic antioxidant in the human erythrocytes (Nakagawa et al., 2008). In another study of various carotenoids, it was observed that the levels of lutein in erythrocytes of Alzheimer's disease patients were significantly lower than those of normal individuals (Kiko et al., 2012). Consumption of lutein capsules enhances lutein concentrations and suppresses phospholipid hydroperoxide formation in human erythrocytes (Nakagawa et al., 2008), so diet consumption of lutein may play a role in antioxidant defense in erythrocytes and therefore develop positive impacts in Alzheimer's disease patients. Since labeling of *Chlorella* products D and M reveals that the products contain no lutein, this implies that *Chlorella* products D and M contain significant levels of lutein (200 mg/100 g dw). The randomized, double‐blind, placebo‐controlled clinical trial was performed by Miyazawa et al. (2013) to investigate the impact of *Chlorella* (8 g *Chlorella*/day/person, which is 22.9 mg lutein/day/person) on PEP and lutein in erythrocytes. They found that 2 months of *Chlorella* supplementation resulted in the amplification of erythrocyte lutein by 4.6 fold, but the tocopherol levels remained unchanged. It appeared that a daily dose of *Chlorella* may help enhance and/or maintain the erythrocyte antioxidant profile and the lutein levels in human beings (Miyazawa et al., 2013). Based on these findings, the consumption of *Chlorella* results in the proper erythrocyte function and has positive outcomes on Alzheimer's disease‐associated dementia in humans.

Major depressive disorder (MDD) is highly prevalent and significantly reduces the quality of life of people. At least once in their lives, a quarter of people have at least one episode of depression. While antidepressants that can be used to treat depression are many, a significant percentage of patients do not conform to the drugs, and some even have side effects (Cui et al., 2024). Consequently, any other types of antidepressant drugs that can effectively manage the disorder and, at the same time, do not possess adverse effects on patients are required. The efficacy of the dried *C. vulgaris* extract supplement (1.8 g/day) for 6 weeks was assessed in patients diagnosed with MDD. The specifics of the symptoms in the participants that improved after treatment included physical and cognitive symptoms of depression (Panahi et al., 2015). Since increased oxidative stress is one of the pathogenetic factors of major depressive disorder, major depressive disorder has been successfully treated with antioxidant therapy (Valko et al., 2007). Such findings would indicate that the generic impact of *Chlorella* supplementation, as a therapeutic, could be due to the role of its antioxidant nutrients and compounds (Vijayavel et al., 2007).

#### 4.2.2 Immune system support

Hasegawa et al. (1999) described the impact of a *Chlorella* (*C. vulgaris*) hot‐water extract on an antigen‐specific response in mice. The 2% *Chlorella* hot‐water extract diet or control diet without *Chlorella* extract was provided to mice for 2 weeks, and then an intraperitoneal injection of casein or complete Freund's adjuvant was conducted. As for the results, there was inhibition of IgE production and level of interleukin‐6 in the mice after the hot‐water extract treatment. They also showed a dose‐dependent up‐regulation of interleukin‐12 and γ‐interferon mRNA, which enhanced the type 1 helper T‐cell immunity and reduced the type 2 helper T‐cell immunity. Based on these findings, *Chlorella* hot‐water extract may have the potential for the prevention/amelioration of type I allergic reactions involving mostly the type 2 helper T lymphocytes.

Allergic disease is a common atypical state of the immune response to various environmental proteins or antigens. CD4^+^ T cells specific to the allergen, which is implicated in starting allergic reactivity, can differentiate into either type 1 or type 2 helper T cells (Miura et al., 2020). CD4^+^ T cells can also be differentiated into type 1 helper T cells when stimulated in the presence of interleukin‐12 and γ‐interferon. On the other hand, interleukin‐4 enhances the differentiation of type 2 helper T cells and suppresses the emergence of type 1 helper T cells (Cenerenti et al., 2022). The type 1 and type 2 helper T cells reciprocally control each other's activity; interleukin‐12 not only induces the type 1 helper T cell response but also controls the type 2 helper T cell response. Interleukin‐12 is an efficient inhibitor of IgE synthesis as it inhibits the generation of type 2 helper T‐cells. Specific IgE of allergens leads to the pathogenesis of allergic disorders (Van Den Eeckhout et al., 2021).

To explain how *C. pyrenoidosa* hot‐water extract can have immunomodulatory effects, polysaccharides soluble in water were extracted, and the composition and structure were analyzed by Hsu et al. (2010). Using GC‐MS, Hsu et al. (2010) found that the major soluble monosaccharide compositions include rhamnose (31.8%), glucose (20.4%), galactose (10.3%), mannose (5.2%), and xylose (1.3%). These soluble polysaccharides were intraperitoneally injected (100 mg/kg body weight) into 6‐ to 8‐week‐old mice. Following 24 h, lipopolysaccharide was regarded as an antigen to mice, and afterward, the serum was harvested after 1.5 h (Hsu et al., 2010). The soluble polysaccharides influenced the production of interleukin‐1 β in macrophages through the toll‐like receptor protein kinase signaling pathway. Interleukin‐1 β is one of the inflammatory and host response mediators to infections (Halperin et al., 2003). From these outcomes, it can be proposed that hot‐water‐soluble polysaccharides of *Chlorella* can be used as agent sources for enhancing antimicrobial activity. Halperin et al. (2003) explored the immunomodulatory effect of *C. pyrenoidosa* at a dose of 200 or 400 mg on the response to influenza vaccination. There were, however, changes in the immune response of subjects to the influenza vaccine after the 28‐day *Chlorella* supplementation, especially in the age group of 50–55 years old.

Salivary secretory immunoglobulin A (SIgA) is the major immunoglobulin involved in the mucosal immunity of humans and may be described as the first line of defense at the mucosal surfaces against pathogens (Y. Li et al., 2020). To establish whether *Chlorella* supplementation enhances the salivary SIgA output in humans, a triple‐blind randomized crossover clinical trial was carried out by Otsuki et al. (2011) on a group of people under *Chlorella (C. pyrenoidosa)* (6 g/day) or placebo intervention for 4 weeks. The trial found no significant change in the salivary SIgA levels before and after placebo administration but a significant increase in the salivary SIgA levels after *Chlorella* administration when compared to the basal level. The overall level of SIgA secretion was also raised with the *Chlorella* supplementation. Based on these findings, it may be concluded that 4‐week *Chlorella* supplementation enhances salivary SIgA levels and enhances host defense status in men.

Natural killer cells are the major element of the innate lymphocyte population that plays a role in antitumor and antiviral immunity (Björkström et al., 2022). To ascertain the impact of the natural food fortification with *Chlorella* on natural killer cells and the first phase of inflammation in people, a randomly controlled, double‐blind clinical trial was performed by Kwak et al. (2012) in healthy adults who ingested *Chlorella* (*C. vulgaris*) in the dosage of 5 g/day or placebo. The *Chlorella* group serum interferon‐γ and interleukin‐1β was significantly higher than the baseline and significantly higher than the control at week 8, and that of interferon‐γ tended to increase. The natural killer cell activities in the *Chlorella* group were higher than in the control group. Therefore, short‐term *Chlorella* treatment has a positive immune‐stimulating effect, which leads to an increase in natural killer activity and the production of interferon‐γ, interleukin‐12, and interleukin‐1 β.

#### 4.2.3 Detoxification effect

Dioxins are chemical byproducts of the family of polychlorinated dibenzo‐p‐dioxin and dibenzofuran that are products of industry and organic pollutants that are easily found in the environment. These compounds are readily and passively diffused in the mammalian gastrointestinal tract and accumulate within the liver, adipose tissue, and breast milk because of their lipophilic character (Ven den Berg et al., 2013). To analyze the behavior of *Chlorella* on fecal excretion of dioxins, Morita et al. (1999) dosed rats with dioxin‐contaminated rice oil. The rats were offered 4 g of a 10% *Chlorella* (*C. vulgaris*)‐containing diet or a control diet that was devoid of *Chlorella* within the 5 days, and the number of fecal dioxins in the rats was determined. The fecal dioxin concentrations were higher in the *Chlorella* group than in the control group. Besides, the result determined that *Chlorella* supplementation can suppress the gastrointestinal absorption of dioxins (by 2%–53%) (Morita et al., 1999). About these results, it can be suggested that *Chlorella* supplementation may be effective in facilitating dioxin elimination.

Heterocyclic amines are now recognized as carcinogenic chemicals that are produced when amino acids, sugars, and creatine in muscle meats such as beef, pork, fish, and poultry react with one another while being cooked at high temperatures (Bito et al., 2020). To determine the impact of *Chlorella* supplement on the detoxification of carcinogenic heterocyclic amines, a randomized, double‐blinded placebo‐controlled crossover trial of *Chlorella* supplement at 100 mg a day for 2 weeks was used by Lee et al. (2015). *Chlorella* supplementation resulted in the urine containing less of the major metabolite of carcinogenic heterocyclic amines, indicating that *Chlorella* may limit the absorption of heterocyclic amines or neutralize the carcinogenic compounds (Lee et al., 2015).

Methylmercury is a neurometabolic form of mercury that arises from inorganic mercury by microorganisms in aquatic ecosystems and through the food chain and is prevalent in fish and shellfish. The main pathway through which people are at risk of being affected by methylmercury is through the consumption of fish (Harding et al., 2018). For example, in many developed countries it is recommended that pregnant women avoid large fish like tuna to prevent fetus exposure to methylmercury (Hsi et al., 2016). Since *Chlorella* enhances the elimination rate of methylmercury and decreases the tissue mercury levels in methylmercury‐fed mice, an open‐label naturalistic trial was carried out by Uchikawa et al. (2010) to assess the effects of *Parachlorella beijerinckii* chlorophyll‐rich preparation (9 g/day) for 3 months on hair and blood mercury levels in healthy subjects. *Chlorella* supplementation has been proven to decrease the amount of mercury in the hair and blood (Maruyama et al., 2018). Like hazardous methylmercury, humans excrete an average of 90% through their feces. Most of the methylmercury present in the liver is exported as a glutathione conjugate through the bile duct with another fraction in the feces. Humans pass more feces due to the existence of dietary fiber in *Chlorella* cells. Cholesterol, protein, and dietary fiber captivate a certain amount of methylmercury in vitro (Bito et al., 2020). Based on these observations, it can be postulated that the tendencies toward the decrease in hair and blood mercury levels in individuals with the use of *Chlorella* may be caused by enhancement of fecal elimination of methylmercury through stimulation of bile secretion, binding of methylmercury with dietary fiber in the ileum, and increased feces production.

Table 2 presents various previous studies exploring the diverse applications and potential of *C. vulgaris* in the food industry, highlighting their results.

**TABLE 2:** Table summarizing previous studies and their results regarding *Chlorella vulgaris* in various applications.

Application	Study	Objective	Results
Food supplement	Belasco (1997), Gouveia et al. (2002), Liu and Chen (2014)	Investigated the use of *Chlorella vulgaris* as an edible, low‐cost, high‐quality food source.	*Chlorella vulgaris* used as a food supplement since World War I, primarily in Germany, China, Japan, the United States, and Europe.
Emulsifier	Raymmundo et al. (2005)	Evaluated the high protein content of *C. vulgaris* for use as an emulsifier.	*C. vulgaris* effectively used as an emulsifier due to its high protein content.
Thickening and gelling agent	Brennan and Owende (2010)	Investigated polysaccharides from *C. vulgaris* for thickening and gelling in foods.	Polysaccharides from *C. vulgaris* used for thickening and gelling in food products.
Natural colorant	Gouveia et al. (2002)	Studied the use of natural colorants from *C. vulgaris* in enhancing food color stability and texture.	Colorants from *C. vulgaris* improved food product color stability and texture.
Bakery products	Graça et al. (2018)	Evaluated the impact of *C. vulgaris* in wheat flour dough on bread quality and rheology.	1–3 g/100 g *C. vulgaris* improved dough rheology; higher amounts negatively impacted texture and bread quality.
Snacks and bars	Shalaby and Yasin (2013)	Investigated the impact of *C. vulgaris* in imitation cheese‐filled croissants.	*C. vulgaris* enhanced sensory quality, improving taste, texture, and overall consumer appeal.
Aquaculture	Ahmad et al. (2020)	Investigated the effects of *C. vulgaris* in aquaculture feeding and disease resistance.	*C. vulgaris* promoted growth, immunity, and stress reduction in fish; higher inclusion rates had negative impacts.
Health benefits and functional properties	Konishi et al. (1985), Tanaka et al. (1990), L. C. Wu et al. (2005), Cha et al. (2008)	Investigated antioxidant and antitumor properties of *C. vulgaris*.	*C. vulgaris* exhibited antioxidant and antitumor activities, with multiple bioactive compounds contributing to health benefits.
	Selkoe (2001), Nakagawa et al. (2008), Kiko et al. (2012)	Explored the impact of lutein from *C. vulgaris* on Alzheimer's disease.	Lutein in *C. vulgaris* may help improve antioxidant defense in Alzheimer's disease patients.
	Miyazawa et al. (2013)	Conducted a clinical trial on the health impacts of *C. vulgaris*.	*C. vulgaris* intake improved health outcomes and decreased disease risk.

### 4.3 Economical and technological aspects

The economic feasibility of *C. vulgaris* as a food replacement lies in its adaptation to be grown in various conditions, namely, freshwater, seawater, and wastewater. This flexibility prevents crowding of agricultural land and water resources, hence making *Chlorella* one of the best natural solutions to the current global food insecurity challenge (Dragone, 2022). Due to its ability to grow extremely fast and produce large yields per area unit, it is more productive than conventional crops in terms of costs in the long run (Gharib et al., 2024). Additionally, technology like photobioreactors, which are the controlled systems for the growth of *Chlorella*, has increased its production capacity. These systems control factors such as light, temperature, and nutrient provision that enhance biomass growth, thus raising biomass yields, whereas contamination threats are avoided (Razzak et al., 2023). The growth of *Chlorella* has also been benefited by these technological improvements in the same respect; the expenses in terms of equipment and facilities are overwhelming for large‐scale production (Bito et al., 2020). However, analyzing overall economic efficiency, *C. vulgaris* is viable for the food industry given its low space requirement, good nutrient recycling, and the possibility of production all over the year.

From a technological point of view, *C. vulgaris* can be presented in various types, in the form of a powder‐like substance, in tablet form, or a liquid extract that can easily be incorporated into foods. The ability to adapt to various formats of food systems makes it suitable to be incorporated into protein supplements, bakery products, beverages, and meat analogues, among others (J. Y. Wu et al., 2023). The improvements in cell disruption technologies, including the use of ultrasonication, enzymatic treatment, and mechanical milling, have boosted the solubility and availability of *Chlorella* nutrients, which is one of the major hurdles to the use of *Chlorella* as food ingredient. However, these technologies help in increasing the processing cost; this factor is being considered in assessing the feasibility of this technology (Canelli et al., 2021).

### 4.4 Toxicological and sensory aspects

Although *C. vulgaris* is considered safe for human consumption, it has the ability to assimilate heavy metals gotten from the environment where it is cultured, and this can cause serious health risks among consumers. *Chlorella* can accumulate heavy metals such as mercury, lead, and cadmium in an uncontrolled environment, and the consumption of *Chlorella* containing such metals is dangerous (Expósito et al., 2021). The application of high measures of quality control is required to have the necessary check for contamination regularly and guarantee that the food products containing *Chlorella* do not pose risks to the consumer's health. Furthermore, the accumulation of heavy metal is a function of the growth medium, and, therefore, the importance of using purified water and controlled nutrient solution in cultivation cannot be underemphasized. Purity standards with reference to heavy metals are relevant and significant, particularly in areas where food safety regulation is high. Special attention has to be paid to the analysis and quality control in order to keep the levels of heavy metals at acceptable level. Additionally, *C. vulgaris* has a hard cell wall and this makes it difficult for the body to metabolize it and therefore affects its safety and effectiveness. Pretreatment may be done mechanically or enzymatically to allow easy digestion of the microalgae and minimize any negative impacts (Weber et al., 2022).

Sensory issues are also experienced with the introduction of *C. vulgaris* into food systems. *Chlorella* has an earthy, strong flavor and smell that is quite unpleasant to some consumer's pallets. This is a limitation, especially when it comes to liberal use in food products, with taste and smell being sensitive factors (Nunes et al., 2023). In order to counter these difficulties, *Chlorella* can be incorporated into foods using different flavor‐masking techniques that include encapsulation and blending *Chlorella* with other strong‐flavored ingredients that can help mask the unique taste and strong sensory properties of *Chlorella* while enhancing its palatability among consumers (Tohamy et al., 2018). The other technique that can be employed is to use *Chlorella* in lesser concentrations so as to preserve the taste of the food supplement while serving the nutritional benefits in the process. Additionally, the investigation of new strains with less intense sensory properties can also help for its use in a wider range of food products (Tohamy et al., 2018).

## 5 CHALLENGES OF *Chlorella* PRODUCTION AND COMMERCIALIZATION

The antibacterial properties of *Chlorella* are strain‐dependent and affected by the growth condition and the extraction procedures. This variability poses a big challenge when it comes to standardization and the consistency in product quality (Hussein et al., 2018). Various species of *Chlorella* contain different biological effects that are determined by their inherited characteristics and growth conditions. For example, light intensity, nutrient supply, and pH may affect the metabolic activities within the cells of *Chlorella* causing fluctuations in the synthesis of bioactive compounds. However, the methods that are used to isolate these compounds from the plant material, such as solvent extraction or supercritical fluid extraction, also impact their quality and activity. Such parameters concern regular and effective antibacterial activity, and their strict regulation implies certain challenges when it comes to mass usage. This challenge requires constant pursuit of knowledge as to the most suitable methods of growing and processing *Chlorella* to develop a set process that can be consistently replicated in subsequent batches.

Cultivation of *Chlorella* is very costly because it has to be grown in a clean environment since it is sensitive to contamination and requires special equipment and sterilization. This makes large‐scale production economically unviable (Rajkumar et al., 2014; Spolaore et al., 2006). A sterile environment is critical as it protects the algae from contamination as well as provides the most ideal conditions for the algae to grow; however, it is capital intensive since one is required to have facilities such as photobioreactors or closed culture systems. Sanitization procedures, required to eradicate undesired microorganisms, are another factor, increasing costs and depth. As with the capital expenditure, specialized equipment for harvesting, drying, and processing of *Chlorella* also enhances the manufacturing cost. Economic constraints within *Chlorella* production are evident as these barriers may hamper the expansion of *Chlorella* production and make it difficult for producers to set affordable prices for its production in the market. To eliminate these challenges, the industry needs to check for affordable technologies and methods of growing to reduce expenses while at the same time enhancing yields and quality.

Increasing *Chlorella* production is problematic since the culture can be easily contaminated and there are variations in the growth rates, and initial investment in the proper structure is needed (Xu et al., 2009). One of the main problems that can be encountered is contamination by another microorganism; this may be fatal to the large production requirement and the setup of large‐scale arrangements. Also, a large amount is usually incurred on maintaining the sterile field and equipment. The rates of *Chlorella* growth fluctuate depending on the existing conditions, and the yields are difficult to predict and to increase constantly. These issues need additional large‐scale photobioreactors and filtration systems to control, which are still considered challenges that add to the complications and the costs of scaling up such processes. The management of these challenges is a never‐ending practice of implementing effective strategies, including adjustment, and employment of sound production mechanisms that are capable of supporting exponential production without contractual innovation on the company's quality and performance.

In commercial preparations, *Chlorella’*s cell wall is not easily digested, and consequently, its nutritional value does not yield as expected (Merchant et al., 2002; Panahi et al., 2016). Due to this, proper methods of processing are required to improve the palatability of this food source for humans. Techniques like mechanical activation, enzymatic degradation, or ultrasonic waves are applied to weaken and increase the permeability of the cell wall, which helps the nutrients to be assimilated by the human body better. However, these processes bring extra costs and issues to the process of production in the organization. Mechanical milling uses mill equipment and is more expensive in energy consumption, and enzymatic treatment uses specific enzymes and can destroy some dangerous compounds. The process of ultrasonication, which is based on the application of sound waves to destroy the cell structures, is also rather precise and costly. An essential issue that is yet unaddressed in the industry includes the proper formulation and processing of *Chlorella* ensuring that it is easily absorbed into the body.

*Chlorella* can accumulate heavy metals from its setting, which is a problem for consumers’ health, hence the need for standardization (Zeraatkar et al., 2016). *Chlorella* contains heavy metals, which include cadmium, lead, and mercury. The capability of extracting those metals makes this algae ideal in bioremediation products but seemingly dangerous if added to the list of foods and nutrient sources. To minimize this risk, producers should have regular monitoring and testing programs to identify the level of heavy metals in the *Chlorella* products. This calls for high analytical procedures like atomic absorption spectroscopy, and or inductively coupled plasma mass spectrometry, which are capital‐intensive processes that have an undesirable impact on the production costs. Sustaining good quality and safe products is vital to customers and fulfilling the regulations, but it also incurs a major cost and opportunity cost to producers.

Some customers complain of the unpleasant taste and smell of *Chlorella*, which reduces the likelihood of food and beverage application (Nakano et al., 2007). However, *Chlorella* has a strong and grassy taste and smell that many consumers could hardly tolerate. There exist certain challenges associated with the use of *Chlorella*, and one of these is that its taste is not very appealing to most users, so producers have to find different ways to mask it, or the product that it will be used in should be one where its taste cannot easily be detected. This can be done by diluting *Chlorella* with flavoring agents in drinking solutions, functional foods such as nutritional bars, or where *Chlorella* has a strong taste, such as in culinary applications that have accompanying tastes that can mask the taste of *Chlorella*. Research in food processing technology like encapsulation or microencapsulation can help conceal taste and smell while retaining the nutritional values. Eradicating these problems is essential for mass consumer acceptance and market penetration for *Chlorella‐based* products.

## 6 FUTURE PROSPECTS FOR *Chlorella vulgaris* IN THE FOOD INDUSTRY

By improving the desired traits and positively influencing traits like nutrient content, growth rate, and environmental adaptability through various genetic manipulation and breeding methods, the Chlorella strain of better qualities can be produced by the scientists. For instance, it could be an enhancement of the protein, vitamin, and essential fatty acid composition of *C. vulgaris* to make it a better supplement. However, to industrialize *Chlorella* production, new and enhanced culture systems that can be used to increase its yield with a reduced combination of costs must be devised. This includes aspects ranging from different choices of bioreactors, adjusting the light and nutrient factors, and enhancing sterile confinements. Information concerning such matters as the differences between open and closed cultivation systems and their effect on productivity and quality will also be highly beneficial to producers. The commonly used methods are mechanical milling, using enzymes to treat the *Chlorella* cell wall, and ultrasonication, which can increase the bioavailability of nutrients to the human body. Such techniques as pulsed electric fields may be more effective and economically viable for increasing digestibility while not adding so much to the production costs.

It will be necessary to conduct a sensory evaluation on encapsulation, mixing, or natural flavorers to mask the *Chlorella*’s unwanted taste. These improvements will be done with the help of sensory analysis and consumer testing of products. Safety measures include the avoidance of heavy metal uptake, cultivation methods, the use of bio‐remedial techniques, and better analytical methods. Solid clinical research interventions can support and substantiate *Chlorella*’s benefits, easing the process of approvals and increasing consumer trust. Reference standards for the production and processing of foods cut across the required level of production, while the reference frame for approval of foods to the market also enhances food safety. This can be done through *Chlorella* smoothies, *Chlorella* snacks, and *Chlorella* supplements to increase its popularity and market sales. Another way for this is through the use of environmental management strategies such as renewable energy, human water conservation and waste management, and economic sustainability. Through improvement of these areas, the possibilities of using *Chlorella* in the food industry can be greatly expanded resulting in improved quality, safety, and sustainability of the products.

## 7 CONCLUSION

*Chlorella vulgaris* has great potential as an influential source of rich nutrition and supplements food shortly. Its high contents of protein, lipids, carbohydrates, vitamins, and minerals augment the nutritive value and applicability of different food items. However, some challenges associated with *Chlorella* include fluctuation in antibacterial activity, high production costs, scaling, and sensory problems. The discovery of *Chlorella* establishes the fact that it possesses numerous qualities that make it an essential food ingredient, among which are antioxidant activity, immune system support, and detoxification. For *C. vulgaris* to become a part of conventional foods, one must maximize the production and processing approaches of the microalgae and enhance the methods on bioreactors and contamination issues. Some customers may have sensory issues that reduce their attraction to the food. However, through flavor‐masking and new food preparations, it may be possible to appeal to them. Stringent measures that befit the regulation of the products should be taken to ensure quality compliance, and testing for contaminants will help in gaining the confidence of the consumers and entry into the market will be achieved. Increase in awareness of the people about the possible health benefits that can be derived from the intake of *Chlorella* and research and development of this chlorophyll‐containing microorganism to be sustainable. In this way, following these measures, *C. vulgaris* could be perceived as a viable, effective, and advantageous food supplement that meets consumers’ requirements for healthy and environment‐friendly foods.

## AUTHOR CONTRIBUTIONS

**Chiao‐An Wang**: Writing—original draft; writing—review and editing. **Helen Onyeaka**: Conceptualization; validation; writing—review and editing; writing—original draft; supervision. **Taghi Miri**: Writing—review and editing; writing—original draft; validation; supervision. **Fakhteh Soltani**: Writing—original draft; writing—review and editing.

## CONFLICT OF INTEREST STATEMENT

The authors declare no conflicts of interest
